# Generating optical superimposed vortex beam with tunable orbital angular momentum using integrated devices

**DOI:** 10.1038/srep10958

**Published:** 2015-07-20

**Authors:** Yu Wang, Xue Feng, Dengke Zhang, Peng Zhao, Xiangdong Li, Kaiyu Cui, Fang Liu, Yidong Huang

**Affiliations:** 1 Department of Electronic Engineering, Tsinghua National Laboratory for Information Science and Technology, Tsinghua University, Beijing, China

## Abstract

An integrated device, which consists of a variable amplitude splitter and an orbital angular momentum (OAM) emitter, is proposed for the superposition of optical vortex beams. With fixed wavelength and power of incident beam, the OAM of the radiated optical superimposed vortex beam can be dynamically tuned. To verify the operating principle, the proposed device has been fabricated on the SOI substrate and experimentally measured. The experimental results confirm the tunability of superimposed vortex beams. Moreover, the ability of independently varying the OAM flux and the geometric distribution of intensity is illustrated and discussed with numerical simulation. We believe that this work would be promising in various applications.

Vortex beams, with an azimuthal component of Poynting vector due to the helical wave front, carry optical orbital angular momentum (OAM). Introduced by Allen in 1992^1^, such beam has a phase component of exp(*jlφ*) and the corresponding OAM of *lћ* per photon, where *l* is the topological charge. As OAM is independent of spin angular momentum (SAM), it provides a new degree of freedom for photons. These features of vortex beams are extremely attractive for applications of optical communication^2^,^3^,^4^, quantum information^5^,^6^,^7^, and particle manipulation^8^,^9^,^10^. Superimposed vortex (SV) beam is the linear superposition of vortex beams with different topological charges. Based on the full understanding of OAM, a growing interest in SV beams could be witnessed recently. The relative amplitudes of vortex beams involved in the superposition could be varied so that more flexibility is obtained. Intuitively, the relative amplitudes could serve as carriers of information in wireless communication^11^. By increasing the number of vortex beams involved in the superposition, it is possible to provide arbitrary OAM *qudit* states for quantum information^12^. Actually, SV beams are also more potential in particle manipulation. Size-selective optical trapping and velocity-tunable optical rotating have been readily achieved^13^,^14^.

For each of the aforementioned applications, a device of SV beams is fundamental and essential. Till now, there are two general approaches reported to generate a SV beam. One is mixing the pre-generated vortex beams together directly^15^, while the other is using a computer-generated hologram screened on a spatial light modulator (SLM)^16^. However, both of the two methods require a supporting optical system with huge footprint, including several optical lenses and apertures. Fortunately, photonic integration could overcome the shortcoming of bulk optics due to the miniaturization and scalability. And it is inspiring to notice the recent progresses on integrated OAM emitters, as several approaches have been reported based on various micro/nano-structures^2^,^4^,^17^. Moreover, the generation of a specific SV beam whose total OAM equals to 0 has been demonstrated using integrated emitters^18^.

Following these previous efforts, an integrated device for the superposition of vortex beams is proposed and demonstrated in this work. This generator consists of a variable amplitude splitter (VAS) and an OAM emitter based on a ring cavity. In the VAS, an incident beam is firstly divided into two beams. By varying the refractive index of silicon, the relative amplitudes of such two beams could be tuned. After injected into the emitter, two collinear vortex beams would be scattered into free space by grating elements, with equal but opposite topological charges. These two vortex beams are coupled into a SV beam naturally. In the experimental work, the proposed device has been fabricated on the SOI substrate with a thermal tuning unit. With comparison between the numerical and experimental results, generation of SV beams with tunable OAM is confirmed. It is also theoretically explained in detail how the dependence of the OAM flux and the geometric distribution of intensity could be successfully decoupled.

## Results

### Principles

**Figure 1:**
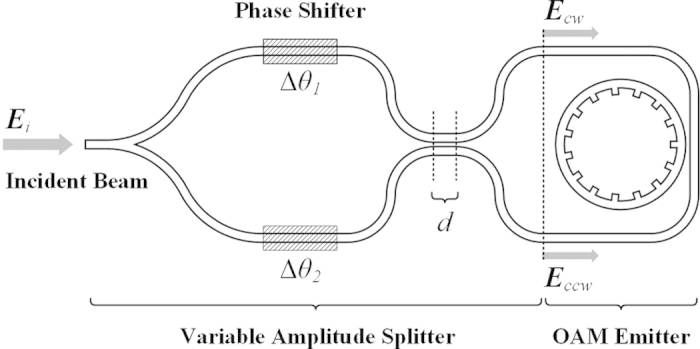
The schematic of the device for the superposition of vortex beams, which consists of a VAS and an OAM emitter.

An integrated OAM emitter has been recently proposed^17^, which is schematically shown in the right part of Fig. 1. The topological charge of vortex beam is determined by the wavelength and the structural parameters of emitter. Actually, the helical directionality of vortex beam, right-handed or left-handed, is also determined by the propagating direction of whispering gallery mode (WGM) involved in the ring cavity. Specifically, counter-propagating WGMs of the same resonant wavelength have exactly inverse phase gradient distributions and would introduce vortex beams with equal but opposite topological charges. Therefore a SV beam could be radiated with two counter-propagating WGMs in the ring cavity. The OAM of the SV beam is a linear combination of that carried by the two vortex beams involved and could be varied by tuning their relative amplitudes^14^. Based on the above concept, an integrated device is proposed for the superposition of vortex beams as shown in Fig. 1, which is consisted of two components, *i.e.*, a VAS and an OAM emitter. It should be mentioned that the propagation mode in the waveguide is only considered as the fundamental quasi-TE mode, which could be excited by controlling the polarization of incident beam.

The VAS is used to split the incident beam into two coherent counter-propagating WGMs in the ring cavity with defined relative amplitudes, namely clockwise beam (***E***_*cw*_, representing the complex amplitude) and counterclockwise beam (***E***_*ccw*_). The VAS consists of a symmetric Y-branch splitter and a directional coupler, with two phase shifters inserted between them. The incident beam is equally divided into two beams after the Y-branch splitter^19^. The phase shifter of each arm afterwards introduces an additional phase shift to the corresponding beam by varying the refractive index of silicon, noted as 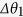 and 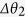. The directional coupler, which is considered as a lossless reciprocal component, then turns the two beams into ***E***_*cw*_ and ***E***_*ccw*_ as follows^20^


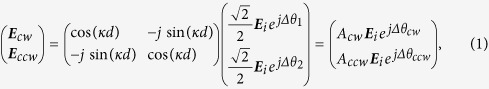


where *d* is the coupling length as shown in Fig. 1 and *k* is the coupling coefficient of two quasi-TE modes in the directional coupler, with real amplitude factors (*A*_*cw*_,*A*_*ccw*_) and phase offsets (Δ*θ*_*cw*_,Δ*θ*_*ccw*_) given by


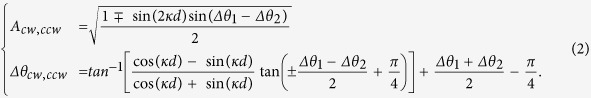


As the transmission and scattering losses are ignored in the VAS, the total power is conserved and given by


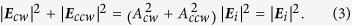


Here, the amplitude ratio of two beams is defined as^14^


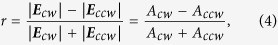


which is within the range of [−1, 1] when sin(2*κd*) = ± 1. With given value of sin(2*κd*), the amplitude ratio *r* is only determined by the introduced phase shift difference, *i.e.*, Δ*θ*_1_−Δ*θ*_2_. By tuning the phase shift difference, two coherent counter-propagating WGMs involved in the ring cavity with defined *r* would be consequently generated after the VAS.

As illustrated in reference^17^, the topological charge *l* of the emitter satisfies


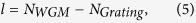


where *N*_*WGM*_ is the azimuthal order of the WGM and *N*_*Grating*_ is the number of grating elements uniformly located around the inner sidewall of the ring cavity. By tuning the corresponding wavelength of the incident beam, the azimuthal order of WGM is varied and then the topological charge of vortex beam could be properly set. In reference^17^, only one incident beam (*E*_*ccw*_) is considered. In our device, however, two counter-propagating incident beams are considered at both ports of the bus waveguide simultaneously. ***E***_*cw*_ would introduce vortex beam with equal but opposite topological charge with respect to that of ***E***_*ccw*_. Therefore, the topological charges of the vortex beams introduced by***E***_*cw*_ and ***E***_*ccw*_ are notated as *l*_*cw*_ and *l*_*ccw*_ with the relation.


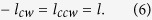


Once the wavelength *λ* and power of incident beam is given, the scattering power *P*_*scat*_ for either ***E***_*cw*_ or ***E***_*ccw*_ is identically the same due to the symmetry of OAM emitter. After scattered by the grating elements, the OAM carried by the SV beam per unit time could be expressed as


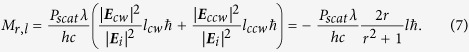


In equation (7), the term of 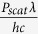 represents the number of photons. For the certain case where there is only one single photon within the SV beam 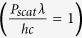, the amplitude ratio *r* provides the probability of the photon with measured value of −*lћ* or *lћ* when one tries to obtain its OAM. At the macroscopical scope with a great amount of photons 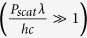, however, equation (7) clearly states the fact that the result of total OAM divided by the number of photons is a continuous or ‘analog’ variable quantity that takes a value anyway between −*lћ* and *lћ*. Consequently, once the wavelength and power of incident beam is given, the OAM of SV beam is only determined by *r*.

Till now, the operating principle of our proposed device for SV beams has been demonstrated. By varying *r*, the OAM of SV beam could be tuned. According equation (2) and equation (4), varying *r* could be readily achieved by tuning the phase shift difference Δ*θ*_1_−Δ*θ*_2_ with thermal-optic effect or plasma dispersion effect^21^.

## Characteristics of radiated SV beams

**Figure 2:**
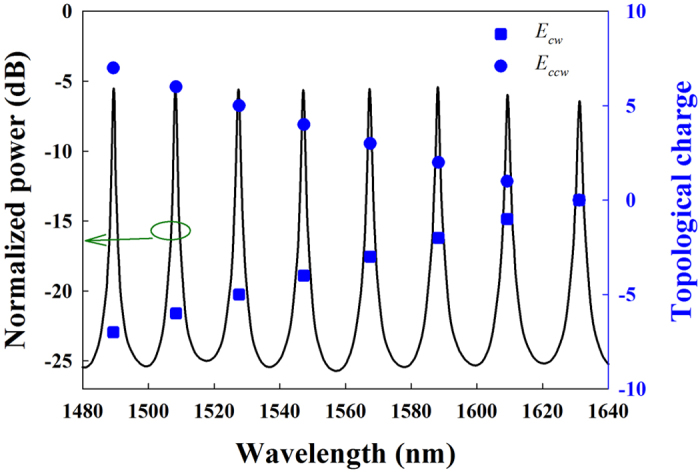
The power spectrum (solid line) of the emitter. The solid squares and the solid circles are the topological charges of vortex beams introduced by ***E***_*cw*_ and ***E***_*ccw*_, respectively.

The inputs to the OAM emitter are analytically based on equation (1) and therefore a single FDTD simulation suffices to determine the characteristics of radiated SV beams. Fig. 2 shows the upward (+*z*) power spectrum (solid line) of the emitter. This spectrum is obtained by normalizing the power flow with respect to the input of ring cavity at one direction (***E***_*cw*_ or ***E***_*ccw*_), representing the emission power conversion efficiency from the incident beam to the radiated beam. According to equation (5), by visually counting the azimuthal order of WGM, the topological charges of vortex beams introduced by ***E***_*ccw*_ are shown by solid circles. The topological charge of ***E***_*cw*_ introduced vortex beam is equal but opposite to that of ***E***_*ccw*_ at the same resonant wavelength, as mentioned in equation (6). Thus the topological charges of vortex beams introduced by ***E***_*cw*_ are shown by solid squares in Fig. 2.

**Figure 3:**
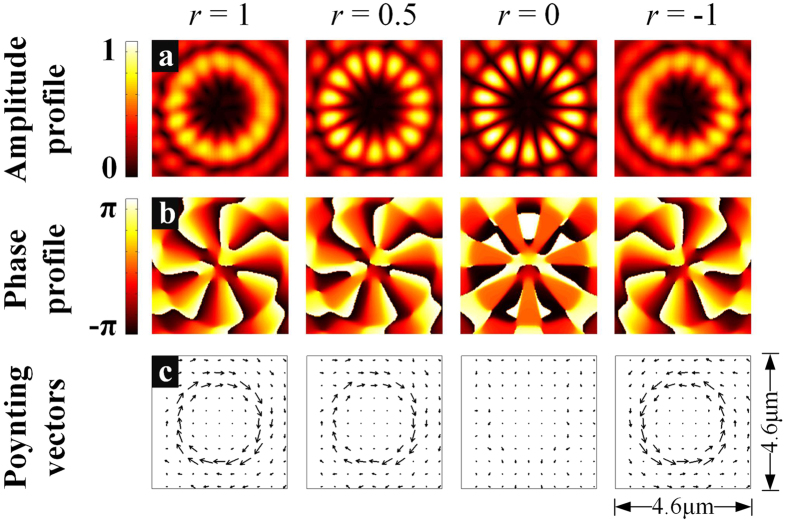
The amplitude and phase profiles of radial components of radiated SV beams when *l* = 7. (**a-b**) (**c**) The corresponding Poynting vectors of radiated SV beams.

By choosing the wavelength of incident beam at a certain resonant peak, a SV beam would be generated with two corresponding opposite topological charges. As a concrete example, the wavelength of incident beam is selected as *λ* = 1490.61*nm* and thus *l* = 7. Fig. 3 (a–c) shows the amplitude and phase (the argument extracted from the complex amplitude) profiles of the radial components and the corresponding Poynting vectors of radiated SV beams. The lengths of Poynting vectors, which representing the magnitudes of energy flux in the focus plane, are normalized with the same factor for easier observation.

For *r* = 1, the OAM of any single photon is −7*ћ* if measured, which can be confirmed from the phase profile. In the first figure of Fig. 3(b), the phase profile is smoothly varied |*l*| times from −*π* to *π* with azimuthal angle and the rotating direction is clockwise as shown by the Poynting vectors in the first figure of Fig. 3(c). For *r* = −1, however, the corresponding OAM of any single photon is 7*ћ* and the SV beam would rotate counterclockwise along the azimuthal direction. For both two cases, the SV beam is actually a pure vortex beam with well-defined topological charge, which is consistent with equation (7).

For the general cases of *r*≠ ± 1, the SV beam is actually composed of two pure vortex components. Here, two concrete cases are shown. For *r* = 0, the two components with inverse rotating direction have equal amplitudes so that the SV beam would be standing wave along the azimuthal direction and there are 2|*l*| bright radial intensity fringes distributed around the center as shown in the third figure of Fig. 3(a). From the corresponding phase profile, it could be found that the phase variation is almost binary along the azimuthal direction. It is actually similar as a cogwheel in mechanics and therefore named as cogwheel beam^13^. The clockwise and counterclockwise Poynting vectors have the same amplitudes so that the total Poynting vectors are completely destructive as shown in the third figure of Fig. 3(c). For *r* = 0.5, the clockwise component is dominant, though partially interfering with the counterclockwise component. The rotating direction of the SV beam, which could be observed through the Poynting vectors, is determined by the dominant vortex component involved as propagating standing wave. Unsurprisingly, the amplitude and phase profiles are between the cases of *r* = 1 and *r* = 0.

Generally, the contrast of amplitude profile is increasing as |*r*| approaching to 0 from 1, resulting in bright radial fringes to the originally blurred profile. Simultaneously, the phase profile is sharpened from a smoothly varying helical distribution to a binary one, losing its helicity gradually. Meanwhile, the original Poynting vectors are decreasing while the counter rotating Poynting vectors are increasing, thus the total Poynting vectors are decreasing.

Although the wavelength of incident beam is selected as *λ* = 1490.61*nm* with *l* = 7, it is of no surprise that the characteristics of SV beams would be quite similar for situations of other resonant wavelengths since only *l* would be varied.

## Measuring setup and experimental results

**Figure 4:**
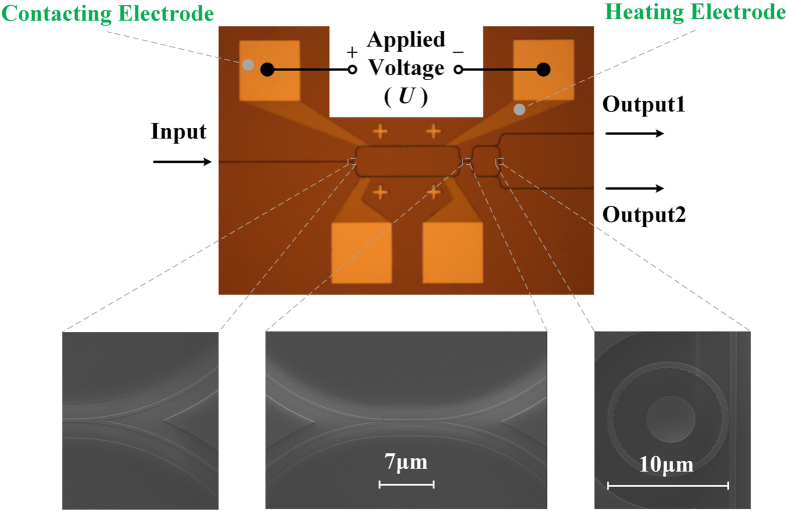
The top view of our fabricated device.

To verify our proposed idea, several samples have been fabricated on the SOI substrate with thermal tuning units. A thermal tuning unit is consisted of a contacting electrode and a heating electrode. The details of device fabrication are presented in the method section. The top view of our fabricated device is demonstrated in Fig. 4. It is worth to notice that two additional output waveguides are introduced to monitor the alignment between the lensed fiber and the tested sample. The driven voltage is applied upon the contacting electrode directly by two metal probes. The underlying heating electrode is used to heat the upper waveguide branch in the VAS with a direct current, leading to an additional phase shift.

Since the quasi-TE mode is excited in the waveguide, the emitted SV beams are azimuthally polarized^17^. An effective method to identify the value of the topological charge of such vectorial beam is by interfering with a coherent right-hand circularly polarized (RHCP) or left-hand circularly polarized (LHCP) beam. The interference profile has a spiral pattern of *l* – 1 (RHCP) or *l* + 1 (LHCP) arms, respectively.

**Figure 5:**
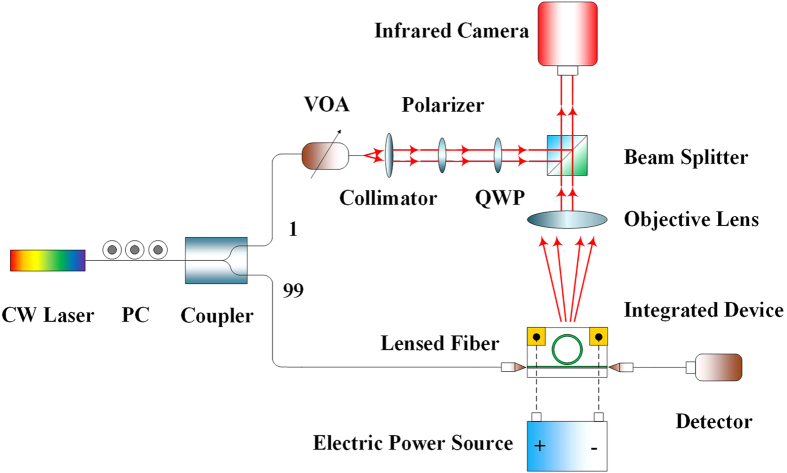
The schematic of our measuring setup.

Figure 5 shows the measuring setup. A polarization controller (PC) subsequently after a continuous-wave laser (CW laser) is applied to ensure a quasi-TE mode excitation in the waveguide of the integrated emitter. Then the beam with desired polarization is divided into two beams by a coupler with the energy ratio of 99:1. The larger one is connected to the lensed fiber and then coupled to the integrated device, while the smaller one is tuned into a RHCP or LHCP planar beam with a collimator, a polarizer, and a quarter-wave plate (QWP). Due to the large coupling losses between the lensed fiber and the waveguide, more power is coupled to the fabricated device deliberately as described above, meanwhile a variable optical attenuator (VOA) is used to balance the energies of the two interfering paths additionally. The phase shift difference Δ*θ*_1_−Δ*θ*_2_ is achieved by thermal-optic effect in the experiment. A driven voltage is applied upon the two ends of contacting electrodes on the integrated device by an electric power source.

**Figure 6:**
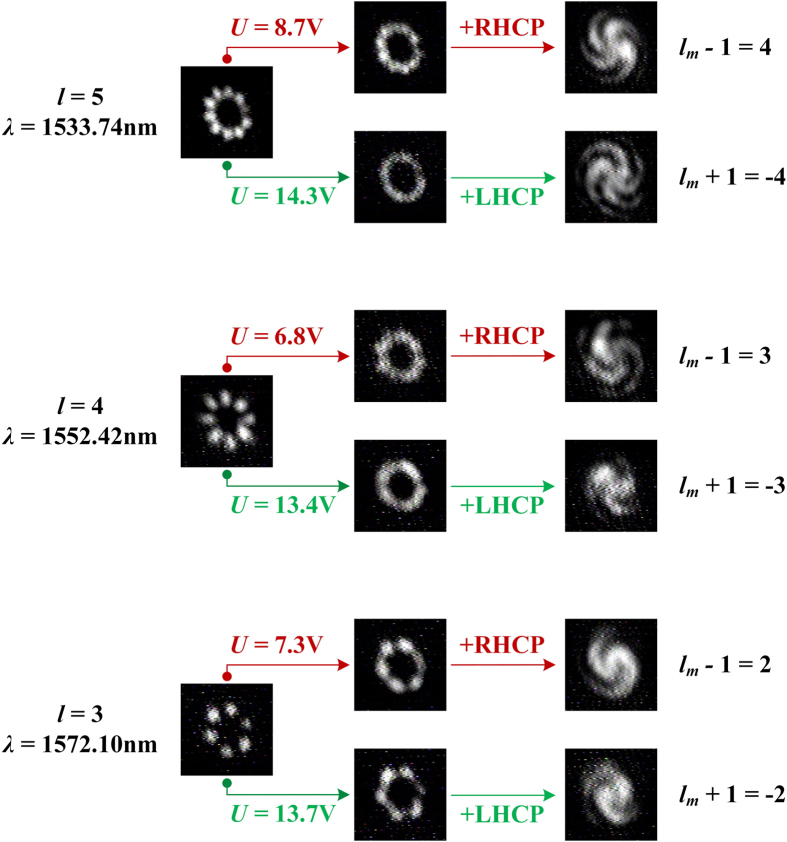
The measured intensity profiles of original SV beams (first column), SV beams with applied voltage (second column), and the interference patterns with a RHCP or LHCP beam (third column). The profiles are obtained with resonant wavelengths of *λ* = 1533.74 nm, 1552.42 nm, and 1572.10 nm, respectively.

Three resonant wavelengths (*λ* = 1533.74 nm, 1552.42 nm, and 1572.10 nm) are selected and the corresponding topological charge is *l* = 5, 4, and 3 according to the results in Fig. 2. The measured results are summarized in Fig. 6 and there are three columns. The first column shows the original profiles without applying voltage. The SV beam indeed has 2|*l*| bright radial intensity fringes distributed around the center as standing wave along the azimuthal direction, which is corresponding to the case with the amplitude ratio of *r* = 0. With relatively low applied voltages (8.7 V, 6.8 V and 7.3 V), the intensity profiles are blurred to become rings as expected and shown in the second column. Each SV beam is tuned to be propagating wave along the azimuthal direction. After interfering with the RHCP beam, the measured value of the topological charge of each SV beam is verified to be 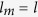 by the interference profiles as shown in the third column. According to equation (7), these are for the case with the amplitude ratio of *r* = −1. With relatively high applied voltages (14.3 V, 13.4 V and 13.7 V), the intensity profiles are blurred again. But the measured topological charge for this case is verified to be *l*_*m*_ = −*l* by the interference profiles with the LHCP beam. The corresponding amplitude ratio is *r* = 1. All these results indicate that the OAM of the SV beam could be dynamically tuned by varying the amplitude ratio *r* of incident beam.

## Discussion

Considering a pure vortex beam radiated from the OAM emitter introduced by ***E***_*ccw*_, the corresponding OAM is *lћ* per photon. The WGM order, in consequence of wavelength, would determine the topological charge *l*. Thus tuning the wavelength of incident beam could vary the OAM of the pure vortex beam and it is also the only method actually. The radius of a pure vortex beam is linear proportional to the absolute value of topological charge^22^ so that the wavelength determines both the OAM and the beam radius, *i*.*e*., the geometric distribution of intensity. Thus, the variation of OAM carried by a pure vortex beam is inevitably accompanied with varied geometric distribution of intensity.

**Figure 7:**
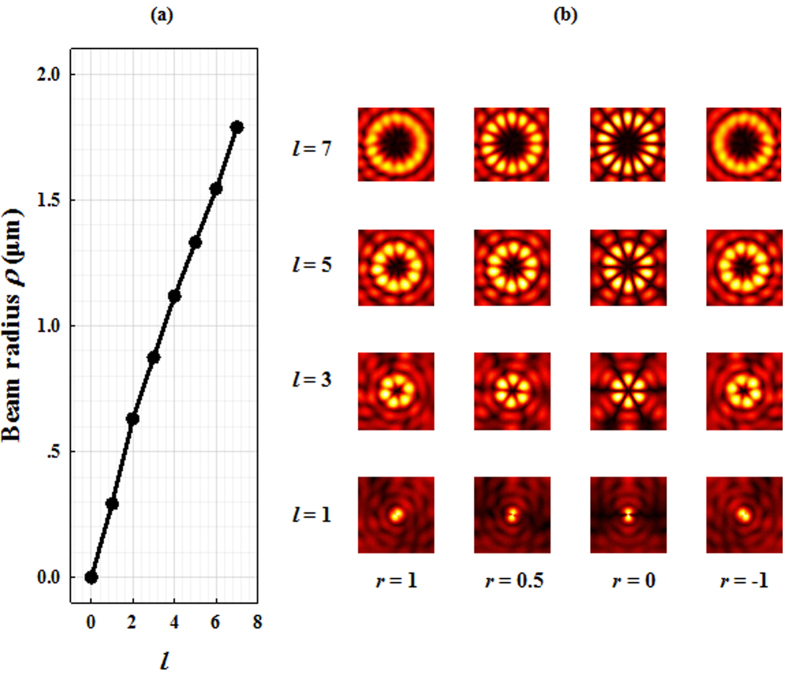
The beam radius *ρ* of radiated SV beam versus *l* . (**a**) (**b**) The amplitude profiles of radial components of radiated SV beams with *l* = 1, 3, 5, and 7.

Fortunately, there is no such limitation for the generated SV beams. A radiated SV beam is composed of two concentric collinear beams with equal but opposite topological charges so that the radius of the SV beam is independent of *r* and only determined by *l*. Fig. 7 shows the relation of the beam radius (location of intensity maxima) of SV beam and *l*. The amplitude profiles of radial components of radiated SV beams with *l* = 1, 3, 5, and 7 are also presented for more clarity. It is obvious that the beam radius is only determined by *l* and is independent of *r*. This characteristic of SV beams could also be obtained from the experiment results shown in Fig. 6.

To analyze the OAM carried by SV beam, OAM flux is numerically calculated. The OAM flux of a SV beam, which is the flow of OAM across the focus plane, could be derived as^23^


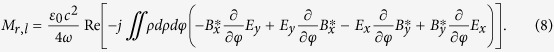


**Figure 8:**
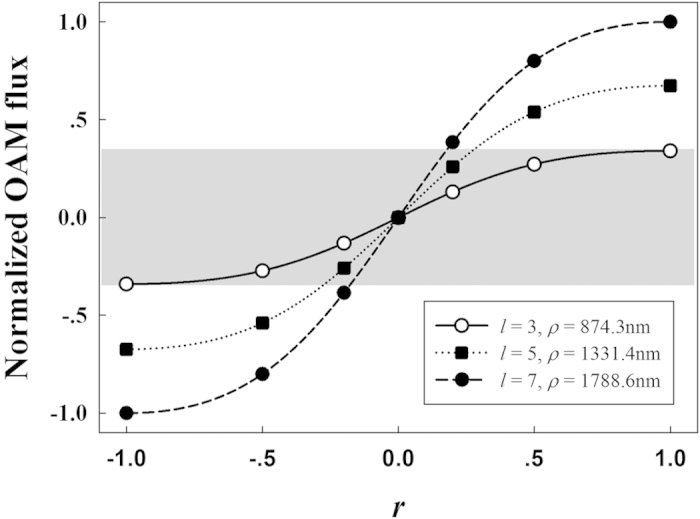
The normalized OAM flux*M*_*r,l*_*/M*_*r = 1,l = 7*_ is tunable by varying *r*, with *l* = 3, 5, and 7 respectively. Different *l* corresponds to different beam radius *ρ*, *i.e.*, different geometric distribution of intensity. With *l* = 3, 5, and 7, the numerical results are shown by discrete hollow circles, solid squares, and solid circles, meanwhile the predictions are shown by the solid, dotted, and dashed lines, respectively.

For certain power of incident beam, the calculated OAM flux is normalized as *M*_*r,l*_/*M*_*r = *1*,l = −*7_ to describe the evolution of OAM with varied *r* and *l*. As a concrete example, *r* = ±1, ±0.5, ±0.2,0 and *l* = 7 are firstly considered. The numerical results on the basis of equation (8) are shown by the discrete solid circles in Fig. 8. Meanwhile, according to equation (7), the normalized OAM flux with *l* = 7 could be deduced as:


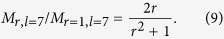


The prediction by equation (9) is also shown by the dashed line for comparison. The numerical results are consistent with the prediction of equation (9) and the OAM flux of SV beam could be continuously tuned in the range from −*M*_*r = *1,*l = *7_ to *M*_*r = *1,*l = *7_ by varying *r*. Moreover, the normalized OAM flux is also numerically calculated for *l* = 3 and 5. The numerical results on the basis of equation (8) with *l* = 3 and 5 are shown in Fig. 8 by discrete hollow circles and solid squares, respectively. Similar to equation (9), continuously varied *r* (−1~1) is also considered for *l* = 3 and 5 according to equation (7). The predictions could be deduced as


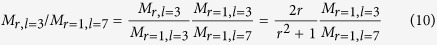


and


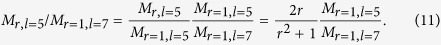


The predictions with *l* = 3 and 5 are also shown in Fig. 8 by the solid and dotted lines, respectively. Without any surprise, the numerical results match the predictions indicated by equation (10, 11) perfectly. The normalized OAM flux could be tuned by varying *r* for all cases of *l* = 3, 5, and 7.

More importantly, there is a gray zone in the figure. Within this zone, for any desired value of the OAM flux from −*M*_*r = *1,*l = *3_ to *M*_*r = *1,*l = *3_, three different radius (874.3 nm, 1331.4 nm, and 1778.6 nm) could be freely selected. That is to say that the geometric distribution of intensity can be independently selected with any desired OAM flux in the gray zone. On the other hand, for any one of the three radiuses, it is also available to continuously tune the OAM flux in the gray zone. Thus, the OAM flux can be independently tuned with defined geometric distribution of intensity. Above all, for a SV beam with given power of incident beam, the OAM flux in the gray zone and the geometric distribution of intensity could be independently selected. However, it is worth to notice that this principle only works efficiently in a certain range (the gray zone in Fig. 6 for example). It is due to the fact that the OAM variation range of a radiated SV beam with certain *l* is limited, as shown by equation (7).

In conclusion, generating SV beam with tunable OAM is demonstrated both in simulation and experiment. As a concrete example, with *l* = 3, 5, and 7, the beam radius (874.3 nm, 1331.4 nm, and 1778.6 nm) and the OAM flux (from −*M*_*r = *1,*l = *3_ to *M*_*r = *1,*l = *3_) could be independently selected. The strong dependence between the OAM flux and the geometric distribution of intensity is successfully decoupled.

We believe that this special characteristic may open up the opportunities for a wider range of applications. For example, it would be easy to integrate this compact device into the microfluidic experimental platform for particle manipulation. The OAM determines the rotating velocity of particles due to the scattering force and the geometric distribution of intensity determines the area where the optical gradient force, which leads to the optical confinement, exists^10^. Thanks to the result that the relation between the OAM and the geometric distribution of intensity is decoupled, the ability and flexibility of manipulation would be dramatically enhanced.

## Method

### Simulation detail

The finite difference time domain (FDTD) method is employed to numerically calculate the performance of our proposed device. The width and the height of waveguides are 220 nm and 500 nm, respectively. The outer radius of ring cavity is 5 μm and the gap distance between the bus waveguide and ring cavity is 60 nm. 43 grating elements are located around the inner sidewall of the ring cavity, exceeding 100 nm from the surface. The width of the elements is as well as 100 nm.

The power spectrum in Fig. 2 is obtained through a circular plane with radius of 5.5 μm, which is 1 μm above the emitter. The amplitude profiles, phase profiles and the Poynting vectors in Fig. 3 and Fig. 7 are obtained from a focus plane 2.5 μm above the emitter. The original data for OAM flux calculation in Fig. 8 is also obtained from the same focus plane.

## Fabrication and measurement detail

The integrated device is fabricated on a 220 nm SOI chip. The structure of the emitter is the same with the simulation described above. The coupling length of the direction coupler in the VAS is 7 μm with a gap of approximate 50 nm. After EBL and ICP, a 600 nm-thick layer of 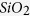 is developed on the top layer by PECVD as protection. The heating electrode of titanium is firstly added on the 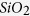 layer by vacuum evaporation, with the thickness of 100 nm. Subsequently, the contacting electrode of aluminum is then added, with the thickness of 300 nm. The section of heating electrode which is right upon the upper waveguide branch is 8 μm wide and 500 μm long. The total resistance of the thermal tuning unit is around 3000 Ω.

The CW laser involved is Santec TSL-210 F and the detector is Agilent 8163 A. The objective lens (Nikon T Plan) is of 50x magnification, with a 2.2 cm super-long working distance. The infrared camera is provided by Electrophysics with the model of 7290 A and the electric power source is Agilent B2901A.

## Additional Information

**How to cite this article**: Wang, Y. *et al.* Generating optical superimposed vortex beam with tunable orbital angular momentum using integrated devices. *Sci. Rep.* **5**, 10958; doi: 10.1038/srep10958 (2015).

## References

[b1] Allen L., Beijersbergen M. W., Spreeuw R. J. C. & Woerdman J. P. Orbital angular momentum of light and the transformation of Laguerre-Gaussian laser modes. Phys. Rev. A 45, 8185–8189 (1992). 9906912 10.1103/physreva.45.8185

[b2] Zhang D., Feng X. & Huang Y. Encoding and decoding of orbital angular momentum for wireless optical interconnects on chip. Opt. Express 20, 26986–26995 (2012). 23187554 10.1364/OE.20.026986

[b3] Huang H. *et al.* 100 Tbit/s free-space data link enabled by three-dimensional multiplexing of orbital angular momentum, polarization, and wavelength. Opt. Lett. 39, 197 (2014). 24562105 10.1364/OL.39.000197

[b4] Fontaine N. K., Doerr C. R. & Buhl L. Efficient multiplexing and demultiplexing of free-space orbital angular momentum using photonic integrated circuits. in *Optical Fiber Communication Conference* OTu1I–2 (Optical Society of America, 2012). at< http://www.opticsinfobase.org/abstract.cfm?uri=OFC-2012-OTu1I.2>. Date of access: 04/03/2012.

[b5] Fickler R. *et al.* Quantum Entanglement of High Angular Momenta. Science 338, 640–643 (2012). 23118185 10.1126/science.1227193

[b6] Simon D. S., Lawrence N., Trevino J., Negro L. D. & Sergienko A. V. Quantum key distribution with Fibonacci orbital angular momentum states. *ArXiv Prepr. ArXiv12063548* (2012). at http://arxiv.org/abs/1206.3548. Date of access: 15/06/2012.

[b7] Nagali E. *et al.* Quantum Information Transfer from Spin to Orbital Angular Momentum of Photons. Phys. Rev. Lett. 103, (2009). http://dx.doi.org/10.1103/PhysRevLett.103.013601. 10.1103/PhysRevLett.103.013601 19659145

[b8] Paterson L. Controlled Rotation of Optically Trapped Microscopic Particles. Science 292, 912–914 (2001). 11340200 10.1126/science.1058591

[b9] MacDonald M. P. Creation and Manipulation of Three-Dimensional Optically Trapped Structures. Science 296, 1101–1103 (2002). 12004124 10.1126/science.1069571

[b10] Padgett M. & Bowman R. Tweezers with a twist. Nat. Photonics 5, 343–348 (2011).

[b11] Bouchal Z. & Celechovský R. Mixed vortex states of light as information carriers. New J. Phys. 6, 131–131 (2004).

[b12] Schulz S. A., Machula T., Karimi E. & Boyd R. W. Integrated multi vector vortex beam generator. Opt. Express 21, 16130 (2013). 23842399 10.1364/OE.21.016130

[b13] Jesacher A., Fürhapter S., Bernet S. & Ritsch-Marte M. Size selective trapping with optical ‘cogwheel’ tweezers. Opt. Express 12, 4129–4135 (2004). 19483955 10.1364/opex.12.004129

[b14] Schmitz C. H., Uhrig K., Spatz J. P. & Curtis J. E. Tuning the orbital angular momentum in optical vortex beams. Opt. Express 14, 6604–6612 (2006). 19516840 10.1364/oe.14.006604

[b15] Kapale K. T. & Dowling J. P. Vortex Phase Qubit: Generating Arbitrary, Counterrotating, Coherent Superpositions in Bose-Einstein Condensates via Optical Angular Momentum Beams. Phys. Rev. Lett. 95, (2005). 10.1103/PhysRevLett.95.173601 16383828

[b16] Lavery M. P. J., Speirits F. C., Barnett S. M. & Padgett M. J. Detection of a Spinning Object Using Light’s Orbital Angular Momentum. Science 341, 537–540 (2013). 23908234 10.1126/science.1239936

[b17] Cai X. *et al.* Integrated Compact Optical Vortex Beam Emitters. Science 338, 363–366 (2012). 23087243 10.1126/science.1226528

[b18] Li H. *et al.* On-chip Electrical Modulation of Phase Shift between Optical Vortices with Opposite Topological Charge. in *CLEO: Science and Innovations* SM3G–5 (Optical Society of America, 2014). at http://www.opticsinfobase.org/abstract.cfm?uri=CLEO_SI-2014-SM3G.5. Date of access: 08/06/2014.

[b19] Kuznetsov M. Radiation loss in dielectric waveguide Y-branch structures. Light. Technol. J. Of 3, 674–677 (1985).

[b20] Kogelnik H. & Schmidt R. V. Switched directional couplers with alternating Δβ. *Quantum Electron*. IEEE J. Of 12, 396–401 (1976).

[b21] Reed G. T., Mashanovich G., Gardes F. Y. & Thomson D. J. Silicon optical modulators. Nat. Photonics 4, 518–526 (2010).

[b22] Curtis J. E. & Grier D. G. Structure of optical vortices. Phys. Rev. Lett. 90, 133901–133901 (2003). 12689289 10.1103/PhysRevLett.90.133901

[b23] Barnett S. M. Optical angular-momentum flux. J. Opt. B Quantum Semiclassical Opt. 4, S7 (2002).

